# A commentary on ‘Single-cell and bulk RNA-sequence identified fibroblasts signature and CD8^+^ T cell - fibroblast subtype predicting prognosis and immune therapeutic response of bladder cancer, based on machine-learning’

**DOI:** 10.1097/JS9.0000000000001769

**Published:** 2024-06-04

**Authors:** Zhen Wang, Guolin Zhang, Xiaofeng Liu, Guang Tan

**Affiliations:** a Department of General Surgery, The First Affiliated Hospital of Dalian Medical University; b Department of Orthopedics, The First Affiliated Hospital of Dalian Medical University, Dalian; c Department of Cardiology, The Second Hospital of Dalian Medical University, Dalian, Liaoning, People’s Republic of China

We read with great interest the recent publication by Li *et al*.^1^ in the International Journal of Surgery concerning their research on bladder cancer immunotherapy. Their study presents the development of a fibroblast-related gene index (FRGI) and a novel subtype combining fibroblasts and CD8^+^ T cells, which they propose as predictive markers for prognosis and therapeutic response in bladder cancer. The authors’ innovative approach offers a comprehensive framework for personalized treatment strategies by integrating diverse biological mechanisms, immune system interactions, and epigenetic features. This significant contribution underscores the potential of tailoring immunotherapeutic interventions based on cellular and molecular signatures. We commend the authors for their groundbreaking work and would like to share some insights and considerations regarding their findings.

We believe that there may be issues with the differential expression analysis of fibroblast-related genes presented in this study. Firstly, for RNA-seq data (TCGA-BLCA), the limma package is designed for normalized data^2^, a detail not addressed in the article. Moreover, Li *et al*.^3^ have reported that for differential expression analysis in population samples, the Wilcoxon rank-sum test is preferred, as limma-voom and other popular algorithms (DESeq2, edgeR, NOISeq, and dearseq) tend to exhibit high false discovery rates. Therefore, it is necessary to reanalyze the data using the Wilcoxon rank-sum test to ensure the robustness and reliability of the findings.

Additionally, the authors constructed a risk model based on the FRGI and clinical variables, showing significant correlations between the risk score and infiltration levels of Tregs and macrophages across multiple bulk transcriptome datasets. However, cell infiltration levels derived from deconvolution algorithms may not be entirely accurate. To address this, the Scissor algorithm developed by Sun *et al*.^4^ could be employed. This algorithm could identify risk-associated subpopulations by integrating bulk and single-cell sequencing data, thereby enhancing the accuracy and validation of the study’s findings.

Moreover, the authors combined FRG characteristics and CD8^+^ T cell activity to construct CD8^+^ T cell-fibroblast subtypes. Patients with high CD8^+^ T cell activity and fibroblast-cold subtype exhibited the best prognosis and higher response rates to immunotherapy, consistent with the findings of Bagaev *et al*.^5^. However, unlike Bagaev’s study, the current study’s subtypes resemble a computational model and have not been validated in real patient cohorts. Therefore, following Bagaev *et al*.’s approach, further investigation and validation based on histological images of patient samples are recommended. Additionally, multiplex immunofluorescence staining and spatial transcriptomics would also be valuable methods for validation.

In summary, we would like to commend Li *et al*.^1^ for their groundbreaking research on the prognostic and therapeutic implications of fibroblast-related gene signatures and CD8^+^ T cell-fibroblast subtypes in bladder cancer. Their work has provided valuable insights into personalized treatment strategies. While acknowledging the significant contributions of their study, we also highlight several methodological concerns and areas for further investigation. We believe that addressing these points will enhance the reliability and applicability of their findings. We eagerly anticipate future studies that build on this important research, offering deeper validation and understanding in clinical settings.

## Ethical approval

Not applicable.

## Consent

Not applicable.

## Source of funding

Not applicable.

## Author contribution

Z.W., G.Z., X.L., and G.T.: conception, design, and writing – original draft; X.L. and G.T.: writing – review and editing; Z.W. and G.Z.: investigation, project administration, and resources supervision. All authors were involved in the final approval of the manuscript.

## Conflicts of interest disclosure

The authors declare that they have no conflicts of interest.

## Research registration unique identifying number (UIN)

Not applicable.

## Guarantor

All authors.

## Data availability statement

Not applicable.

## Provenance and peer review

Not commissioned, externally peer-reviewed.
